# Early Warning System for West Nile Virus Risk Areas, California, USA

**DOI:** 10.3201/eid1708.100411

**Published:** 2011-08

**Authors:** Ryan M. Carney, Sean C. Ahearn, Alan McConchie, Carol Glaser, Cynthia Jean, Chris Barker, Bborie Park, Kerry Padgett, Erin Parker, Ervic Aquino, Vicki Kramer

**Affiliations:** Author affiliations: California Department of Public Health, Richmond, California, USA (R.M. Carney, C. Glaser, C. Jean, K. Padgett, E. Parker, E. Aquino);; City University of New York, New York, New York, USA (S.C. Ahearn, A. McConchie);; University of California, Davis, California, USA (C. Barker, B. Park);; California Department of Public Health, Sacramento, California, USA (V. Kramer)

**Keywords:** arboviruses, birds, disease outbreaks, geographic information systems, insect vectors, mosquitoes, surveillance, West Nile virus, California, research

## Abstract

The Dynamic Continuous-Area Space-Time (DYCAST) system is a biologically based spatiotemporal model that uses public reports of dead birds to identify areas at high risk for West Nile virus (WNV) transmission to humans. In 2005, during a statewide epidemic of WNV (880 cases), the California Department of Public Health prospectively implemented DYCAST over 32,517 km^2^ in California. Daily risk maps were made available online and used by local agencies to target public education campaigns, surveillance, and mosquito control. DYCAST had 80.8% sensitivity and 90.6% specificity for predicting human cases, and κ analysis indicated moderate strength of chance-adjusted agreement for >4 weeks. High-risk grid cells (populations) were identified an average of 37.2 days before onset of human illness; relative risk for disease was >39× higher than for low-risk cells. Although prediction rates declined in subsequent years, results indicate DYCAST was a timely and effective early warning system during the severe 2005 epidemic.

## Abstract 2

TOC Summary: This system effectively identified high-risk human population areas.

West Nile virus (WNV; family *Flaviviridae*, genus *Flavivirus*) is a mosquito-borne pathogen that has led to ≈30,000 reported (>325,000 estimated) human cases and 1,172 reported deaths in the United States since it was first detected in New York, New York, in 1999 (*1*). The virus was first detected in California in a pool of *Culex tarsalis* mosquitoes in July 2003 (*2*), and in 2004 and 2005 the state had the highest number of reported human cases (779 and 880, respectively) and deaths (29 and 19, respectively) in the United States (*3*). Humans are incidental, dead-end hosts of WNV and generally become infected after intense viral amplification and spillover from local avian populations (*4*). Birds are the natural reservoir and amplification hosts of WNV and infections can cause death rates up to 100% among avian species (*5**,**6*). Beginning in 2000, bird carcasses in California were submitted by local agencies to the WNV Dead Bird Surveillance Program (DBSP) at the California Department of Public Health (CDPH; previously known as the California Department of Health Services) as part of the California Mosquito-Borne Virus Surveillance and Response Plan (*7**,**8*). A toll-free telephone hotline and website for recording public reports of dead birds was established in 2002.

Previous efforts for the early detection and monitoring of WNV activity have used dead bird density or spatial scan statistic as a proxy for transmission risk for humans (*9**–**13*). However, aggregation of reports over nonuniform spatial units (i.e., counties and census tracts) may fail to detect WNV amplification clusters that span regional boundaries or that are contained within large areas. In addition, temporal aspects of the WNV transmission cycle should be considered to avoid false-positive identifications in circumstances in which sustained but slow transmission leads to an accumulation of dead bird reports above the designated risk threshold but does not result in spillover to the human population.

Another approach is the DYCAST system (*14**,**15*), implemented in New York, New York, in 2001 and Chicago, Illinois, in 2002. This system detects statistically significant spatiotemporal clustering of dead bird reports by modeling the WNV amplification cycle using biological parameters; it also includes a statistical method for evaluating effectiveness of human case predictions in space and time. Results indicated that clusters of dead bird reports and human cases of WNV were significantly associated in space and time (*15*). This association suggests that this procedure may be useful for predicting areas at high risk for WNV transmission to humans. Because there is no drug prophylaxis, human vaccine, or treatment available for WNV, integrated pest management and personal mosquito protection remain the only options for reducing human illness and death, and early warning of high-risk areas allows for these measures to be implemented in a timely and effective manner. The objective of the present study was to evaluate implementation of DYCAST as an early warning system in California to target public education campaigns, surveillance, and mosquito control efforts during an anticipated statewide outbreak of WNV.

Methods

## Data

Public reports of dead birds were obtained from the DBSP. Through press releases and various types of media campaigns at state and local levels, citizens were encouraged to use the hotline (1-877-WNV-BIRD) and website (www.westnile.ca.gov) to report dead birds (*7*). Information regarding location, date found, and species was collected for each dead bird reported to the hotline; multiple dead birds included in a single report were treated as multiple reports. Hotline staff screened and entered these data into an Access database (Microsoft Corporation, Redlands, WA, USA); data were subsequently geocoded by using ArcMap version 9.1 and associated 2005 StreetMap USA Plus AltNames street dataset (Environmental Systems Research Institute, Inc., Redlands, CA, USA). WNV became a reportable disease in California in 2005, and human data were collected by local health departments by standardized case history forms. Data were subsequently stripped of personal identifiers, and addresses were geocoded by using a CDPH batch geocoding service that used multiple reference databases (www.ehib.org). Use of human data was approved by the institutional review board at the California Health and Human Services Agency (project no. 05-06-51).

## Procedure

**Figure 1:**
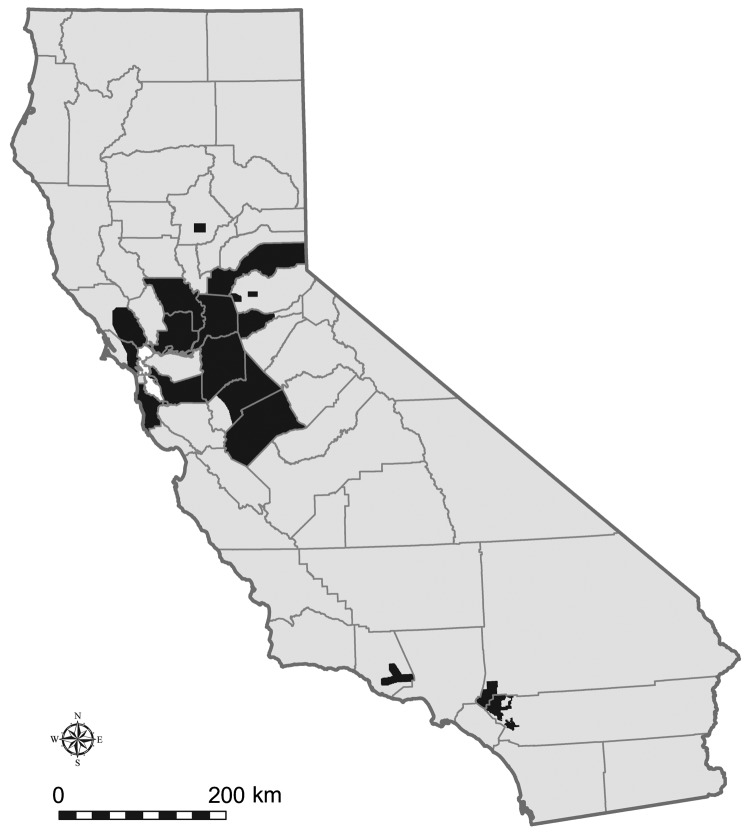
California counties with 2005 Dynamic Continuous-Area Space-Time (DYCAST) analysis regions (32,517 km^2^), shown in black. Data were mapped by using ArcMap version 9.3.1 (Environmental Systems Research Institute, Inc., Redlands, CA, USA) and North American Datum of 1983, High Accuracy Reference Network (NAD83 HARN) California II State Plane coordinate system (Lambert Conformal Conic Projection).

**Figure 2:**
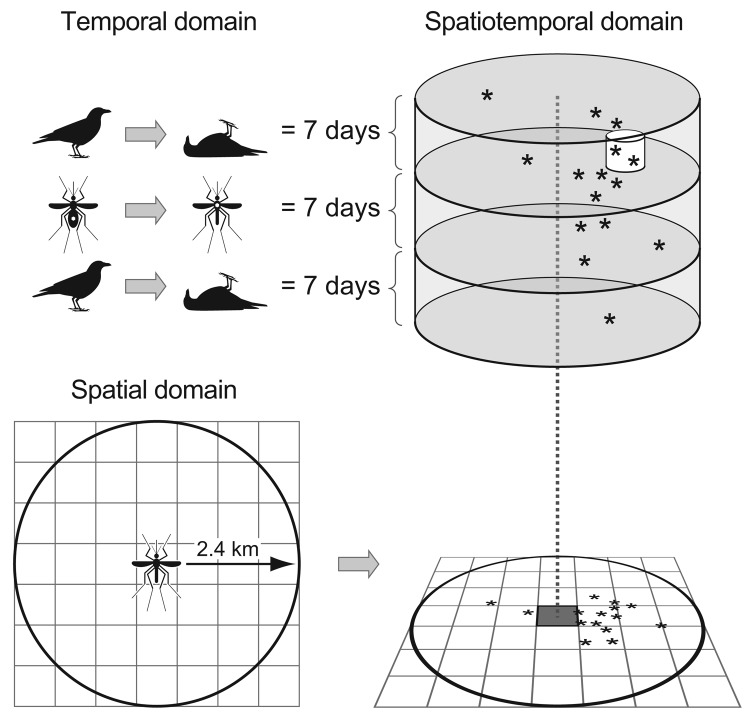
Schematic of the Dynamic Continuous-Area Space-Time (DYCAST) procedure, illustrating domains of Knox test (*16**,**17*) implemented at the center of an individual ≈0.44 km^2^ grid cell. The 2.4-km (1.5-mi) radius of the spatial domain represents twice the daily feeding distance (*14*) of *Culex* spp. mosquitoes in California (*18*) and is equivalent to the effective flight range of these vectors (*19*,*20*). The 21-day temporal domain accounts for the extrinsic incubation period of West Nile virus (*21*) and 2 avian infection cycles of 7 days each (*5**,**14**,**22*). These bounds define the spatiotemporal domain, within which reports of dead birds (asterisks) are evaluated for proximity in space (0.40 km) and time (3 days) (small white cylinder). Statistical significance of dead bird report pairing is assessed by using random simulations (p<0.1) (*15*). Procedure is repeated at other cell centers to create a continuous surface of risk.

The DYCAST procedure was implemented by using GIS software, Smallworld 3.2.1, and Magik programming language (General Electric Company, Fairfield, CT, USA). Regions comprising 32,517 km^2^ among 16 participating agencies in 17 counties were superimposed by grids consisting of ≈0.44 km^2^ (≈0.17 mi^2^) cells (Figure 1). Clustering of dead bird reports was quantified by using a Knox test (*16**,**17*) implemented from the center of individual cells; spatial and temporal parameters were defined by using biologically relevant values (Figure 2). The 2.4-km (1.5-mi) radius of the spatial domain represents 2× the daily feeding distance (*14*) of *Culex* spp. mosquitoes in California (*18*). The effective flight range of these mosquitoes is also 2.4 km (*19*), which corresponds to the maximum distance from breeding sites over which a sufficient number of vectors are able to disperse a mosquito-borne disease (*20*). The temporal domain of 21 days was based on a 7-day extrinsic incubation period of WNV, which ranges from 5 to 8 days for *Culex* spp. mosquitoes at 28ºC in California (*21*), plus 2 avian infection cycles of 7 days each (approximate maximum time from infection to death 5,14,22; ). Candidate values for defining proximity of dead birds in space (0.40, 0.56, and 0.64 km) and time (3, 4, and 5 days) were based on the limited mobility (caused by lethargy, ataxia, and reluctance to fly) and lifespan of infected amplification hosts (*5**,**14**,**23*). During model calibration, locations of human cases were compared with DYCAST risk maps generated by using various combinations of candidate values; 0.40 km (0.25 mi) and 3 days were selected as the optimal combination for the final model. For this calibration, the daily DYCAST procedure was run retrospectively (once during May 2005) by using dead bird and human case data from May 1 through September 30, 2004 within Los Angeles, Orange, Riverside, and San Bernardino Counties, which contained 664 (85.2%) of 779 statewide cases in 2004.

The DYCAST procedure was run at the center of every cell for which a minimum of 15 birds (the analysis threshold) was reported within the spatiotemporal domain, to minimize statistical instability that otherwise occurs at lower numbers of birds (*14*). Clustering was evaluated by comparing the observed number of pairs of dead birds that were close in both space and time (based on aforementioned values of proximity), with the expected number of pairs given a random spatiotemporal distribution of these reports (*15*). The resulting p values were assigned to individual cells, which were considered to indicate high risk for WNV transmission to humans at p<0.1 (*15*).

## Evaluation

Model evaluation was conducted by analyzing the relationship between the location of human cases and the ability of DYCAST to predict their occurrence in both space and time. Prediction was defined as the identification of a cell as high risk before or on the date of illness onset (*15*) of the earliest case located within a cell. Sensitivity was calculated as the number of high-risk cells classified as predicted (true positives) divided by the total number of cells in which a human case occurred. Specificity was calculated as the proportion of low-risk cells without cases (true negatives) to the total number of cells without cases. Because agreement between model predictions and cases can occur by chance, a spatiotemporal implementation of the κ statistic was used to provide a measure of chance-adjusted agreement (*15**,**24*).

## Implementation

An initial pilot phase and subsequent prospective implementation occurred through a cooperative agreement with the Center for Advanced Research of Spatial Information at Hunter College, City University of New York. The Center for Vectorborne Diseases (CVEC) at the University of California Davis provided server infrastructure (Microsoft SQL Server, Microsoft Corporation; ArcIMS, Environmental Systems Research Institute, Inc.) for data exchange and implementation of interactive online risk maps, in collaboration with CDPH and the Mosquito and Vector Control Association of California. The Center for Advanced Research of Spatial Information calibrated and ran the DYCAST procedure and exported the data to the CVEC map server. During the pilot phase, animations of daily risk from June 1 through June 23, 2005, were retrospectively generated for 3 study areas that were selected based on high numbers of dead bird reports: the south Sacramento Valley region (Sacramento, Placer, and Yolo Counties), the central San Joaquin Valley region (Fresno, Kings, and Tulare Counties), and the greater Los Angeles area.

**Figure 3:**
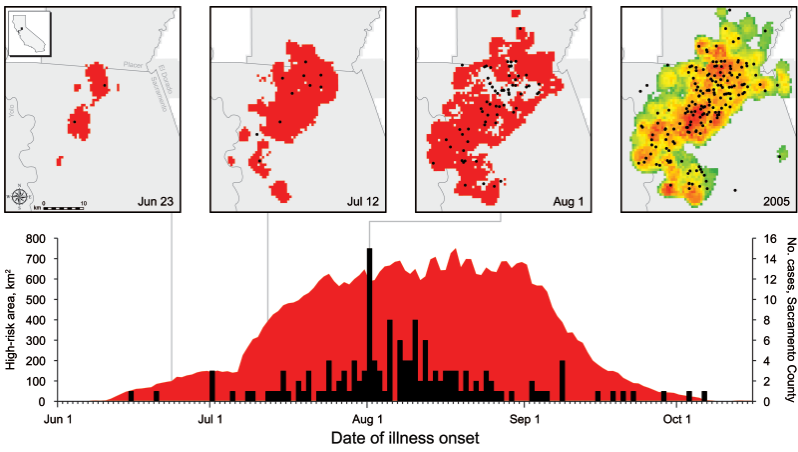
Dynamic Continuous-Area Space-Time (DYCAST) risk maps (top) and timeline (bottom) of West Nile virus epidemic in Sacramento County, California, 2005. Within timeline, black bars represent reported human cases within Sacramento County by date of onset of illness (n = 152; 11/163 cases were missing spatial and/or temporal data), and red region represents total area in Sacramento County designated by DYCAST as high risk by date of analysis. Maps illustrate areas of high-risk (red) cells during the last day of pilot-phase analysis (Jun 23), which detected the 2 emerging clusters seen above in the Arden-Arcade and Citrus Heights regions, as well as during rapid expansion of high-risk areas (Jul 12) and the peak of the epidemic (Aug 1). Map at far right displays cells color coded by number of days designated by DYCAST as high risk during 2005, from green (low) to red (high) (range 1–94 days, mean 47.7 days, median 51 days, SD 19.9 days). Human cases with onset of illness before or including respective date of analysis are shown as black circles; gray area represents DYCAST analysis regions. Inset at top left illustrates location (black square) in California corresponding to the risk maps. Data were mapped by using ArcMap version 9.3.1 (Environmental Systems Research Institute, Inc., Redlands, CA, USA) and North American Datum of 1983, High Accuracy Reference Network (NAD83 HARN) California II State Plane coordinate system (Lambert Conformal Conic Projection).

Prospective modeling began on June 17, and on July 1 the system was fully implemented and integrated into the CDPH WNV Surveillance Program. This implementation involved running the DYCAST procedure for analysis regions every weekday through November 1, 2005; daily risk maps (Figure 3) were generated and made available in real time to mosquito control agencies via the CVEC password-protected website, the California Vectorborne Disease Surveillance Gateway (www.calsurv.org). These interactive maps were overlaid with county boundaries, streets, and locations of reported and WNV-positive dead birds.

## Implementation 2006–2009

Beginning in 2006, DYCAST was implemented for the entire state of California and adopted as a formal component of the California Mosquito-Borne Virus Surveillance and Response Plan (*8*). Addresses of where dead birds were found were automatically geocoded in real time by using the Yahoo Maps application programming interface (Yahoo! Inc., Sunnyvale, CA, USA), which allowed hotline staff to validate location data while callers remained on the line; birds not automatically geocoded were omitted from DYCAST analysis. Interactive DYCAST risk maps were made available online to local mosquito control agencies and integrated with dead bird, mosquito, and sentinel chicken surveillance data from May 1 through October 31, 2006, May 1 through August 31, 2007 and 2008, and March 1 through August 31, 2009. Statewide reports of DYCAST activity, including maps and animations of high-risk areas over time, were sent to local agencies on a routine basis. A real-time alert system was also introduced in 2006 to provide custom DYCAST reports and interpretations for counties experiencing rapidly increasing or elevated levels of high-risk areas (*8*).

In December of 2006 and 2007, links to web-based surveys regarding the DBSP were provided by email to 64 local mosquito control agencies in 47 counties, in part to assess which agencies used DYCAST to assist mosquito larviciding or adulticiding activities each year. For agencies that participated in the 2005 DYCAST program, the 2006 survey also asked if DYCAST results were used “to assist public education or to promote dead bird reporting” in 2005 (control activities were not surveyed for this year). Rate ratios (RRs) were used to compare annual DYCAST prediction rates of reported human WNV cases between agencies that did and did not use DYCAST to assist each mosquito control activity (*25*).

Results

**Figure 4:**
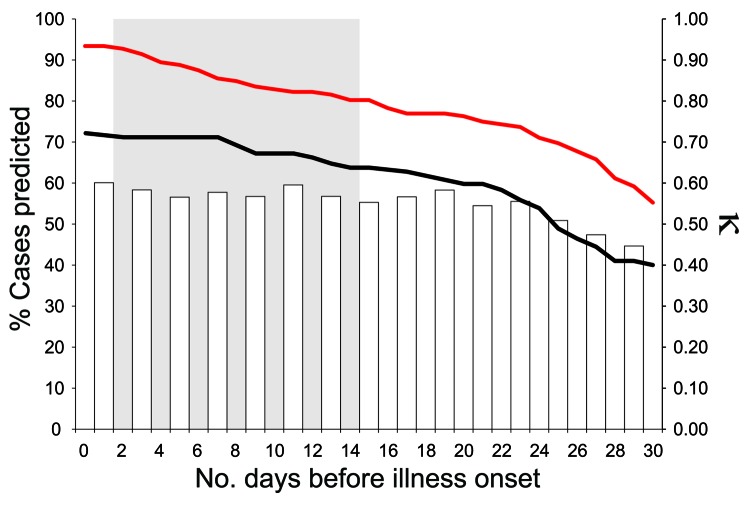
Percentages of reported human West Nile virus cases within Sacramento County (red line) and remainder of analysis regions (black line) predicted by Dynamic Continuous-Area Space-Time (DYCAST) in 2005, as well as κ values (gray bars; calculated every other day with 1-day temporal window *15**,**24*; ) illustrating chance-adjusted agreement between DYCAST results and cases in all analysis regions, by number of days before onset of illness. The wide gray vertical band represents the 2–14 day range of the human West Nile virus incubation period (*28*).

During 2005, a total of 124,876 calls were placed to the DBSP hotline, >3 million hits were made to the website, and 109,358 dead birds were reported in California (Table 1) (*26*). DYCAST identified high risk in 9.7% of the analysis regions (7,160/73,767 cells; 3,139/32,517 km^2^), with cells identified as high risk for a mean total of 39.0 days (range 1–117, median 39, SD 22.8 days). Relative risk of a WNV case in high-risk cells compared with low-risk cells was 39.10 (95% confidence interval [CI] 29.80–51.30; p<0.0001). Sensitivity and specificity of the DYCAST system were 80.8% (269/333 cells) and 90.6% (66,543/73,434 cells), respectively (Table 2). Prevalence of cells containing cases was 0.45% (333/73,767 cells), which resulted in low positive predictive value (3.8%; 269/7,160 cells) and high negative predictive value (99.9%; 66,543/66,607 cells). κ values maintained a moderate strength of chance-adjusted agreement (0.40<κ<0.60; *27*) for >4 weeks before onset of illness (Figure 4). Overall, 289/354 (81.6%) of cases were predicted (Table 3), with cells identified as high risk before onset of illness by a mean of 37.2 days (range 0–126, median 34, SD 20.9 days). A total of 252/354 (71.2%) of cases were predicted 15 days before onset of illness, and >50% of cases (179/354, 50.6%) were predicted 30 days before onset of illness.

**Table 1:** Reported dead bird and human West Nile virus surveillance data, California, USA, 2003–2009*

Surveillance	2003	2004	2005	2006	2007	2008	2009
Dead birds							
Reported	8,650	93,053	109,358	46,365	27,611	33,594	15,472
Tested	1,765	5,723	9,227	6,535	6,000	6,124	2,805
Positive	96	3,232	3,046	1,446	1,396	2,568	515
Humans							
Cases	3	779	880	278	380	445	112
Fatalities	0	29	19	7	21	15	4

*Includes entire state of California. Data sources: California Department of Public Health, Vector-Borne Disease Section (http://westnile.ca.gov) (*2**,**3*).

**Table 2:** Comparison of number of cells that contained reported human West Nile virus cases and number of cells identified as high risk, California, USA, 2005*

High risk	Contained case		Total
	Yes	No	
Yes	269	6,891	7,160
No	64	66,543	66,607
Total	333†	73,434	73,767

*True positive (yes/yes) designates cell identified by Dynamic Continuous-Area Space-Time (DYCAST) model as high risk before or on the date of onset of illness of earliest case located within cell. If cell was identified as high risk after date of onset of illness, or cell was never identified as high risk and a case occurred within it, it was designated false negative (yes/no). †Number of cells that contained cases is less than the number of cases (354) because of 14 cells that contained 2 predicted cases, 3 cells that contained 3 predicted cases, and 1 cell that contained 2 missed cases.

**Table 3:** Reported human West Nile virus cases predicted by DYCAST system by county, California, 2005–2009*

County	No./total cases (%)

2005†	2006	2007	2008	2009
Alameda	NR	0/1 (0)	NR	0/1 (0)	NR
Amador	0/1 (0)	NR	NR	NR	NR
Butte	0/1 (0)	4/29 (13.8)	2/14 (14.3)	0/4 (0)	0/2 (0)
Calaveras	–	NR	NR	0/1 (0)	NR
Colusa	–	0/3 (0)	0/2 (0)	0/1 (0)	NR
Contra Costa	–	4/8 (50.0)	3/3 (100.0)	3/3 (100.0)	0/5 (0)
El Dorado	–	0/2 (0)	NR	0/1 (0)	0/1 (0)
Fresno	–	6/9 (66.7)	9/17 (52.9)	0/2 (0)	2/11(18.2)
Glenn	–	2/10 (20.0)	0/1 (0)	0/1 (0)	NR
Imperial	NR	0/1 (0)	0/2 (0)	NR	NR
Kern	–	2/45 (4.4)	74/126 (58.7)	2/2 (100.0)	0/15 (0)
Kings	–	0/1 (0)	0/6 (0)	0/1 (0)	0/3 (0)
Lake	NR	0/1 (0)	NR	NR	NR
Los Angeles	–	0/12 (0)	2/31 (6.5)	18/147 (12.2)	1/16 (7.1)
Madera	–	NR	0/2 (0)	NR	NR
Marin	NR	0/1 (0)	NR	NR	NR
Mendocino	NR	NR	0/2 (0)	NR	NR
Merced	15/24 (62.5)	0/4 (0)	0/3 (0)	0/1 (0)	0/4 (0)
Mono	NR	0/1 (0)	NR	NR	NR
Monterey	NR	NR	NR	NR	0/1 (0)
Napa	NR	0/1 (0)	0/1 (0)	NR	NR
Nevada	–	0/1 (0)	NR	NR	NR
Orange	–	0/5 (0)	0/9 (0)	18/60 (30)	0/2 (0)
Placer	29/32 (90.6)	3/8 (37.5)	1/4 (25.0)	1/5 (20.0)	NR
Riverside	0/10 (0)	0/4 (0)	0/16 (0)	0/55 (0)	0/2 (0)
Sacramento	142/152 (93.4)	9/15 (60.0)	7/22 (31.8)	4/12 (33.3)	NR
San Bernardino	0/6 (0)	0/3 (0)	0/3 (0)	5/29 (17.2)	0/2 (0)
San Diego	–	NR	0/12 (0)	1/30 (3.3)	0/4 (0)
San Joaquin	25/34 (73.5)	4/8 (50.0)	3/8 (37.5)	3/9 (33.3)	0/8 (0)
San Luis Obispo	NR	0/1 (0)	NR	NR	NR
San Mateo	0/1 (0)	NR	NR	NR	NR
Santa Clara	–	3/5 (60.0)	1/3 (33.3)	0/1 (0)	NR
Shasta	–	2/4 (50.0)	1/9 (11.1)	0/1 (0)	NR
Solano	4/5 (80.0)	6/8 (75.0)	NR	1/1 (100.0)	NR
Sonoma	0/1 (0)	NR	0/1 (0)	NR	NR
Stanislaus	67/79 (84.8)	3/10 (30.0)	7/20 (35.0)	6/16 (37.5)	0/12 (0)
Sutter	–	0/12 (0)	0/2 (0)	NR	NR
Tehama	–	0/6 (0)	0/3 (0)	0/4 (0)	NR
Tulare	–	0/5 (0)	0/9 (0)	2/3 (66.7)	0/3 (0)
Tuolumne	–	NR	NR	NR	NR
Ventura	NR	1/2 (50.0)	0/1 (0)	NR	NR
Yolo	7/8 (87.5)	18/26 (69.2)	0/1 (0)	0/1 (0)	0/2 (0)
Yuba	–	0 (0/3)	NR	NR	NR
Total	289/354 (81.6)	67/255 (26.3)	110/333 (33.0)	64/392 (16.3)	3/93 (3.2)

*DYCAST, Dynamic Continuous-Area Space-Time; NR, no reported human West Nile virus cases; –, nonparticipating counties with reported cases in 2005. Records without a geocodable address or onset of illness cannot be included in the DYCAST model and are therefore not included in these totals. Counties with no reported cases from 2005–2009 are not shown. †Analysis regions (Figure 1) consisted of the 16 agencies that participated in the 2005 DYCAST program. The participating region within El Dorado County had no reported cases in 2005.

According to the 2006 survey, 10/14 (71.4%) of responding local mosquito control agencies within the analysis regions used DYCAST results to assist public education or to promote dead bird reporting in 2005. DYCAST risk maps were also used to direct WNV surveillance (and ultimately control) efforts as early as the pilot phase, when 2 emerging clusters of high-risk cells were identified around the Arden-Arcade and Citrus Heights regions of Sacramento County on June 24, 2005 (Figure 3, June 23, 2005). These results were immediately shared with the Sacramento-Yolo Mosquito & Vector Control District (SYMVCD), which used the risk maps to deploy mosquito traps within the 2 high-risk clusters on June 28 (D. Brown, SYMVCD, pers. comm.). On June 29, SYMVCD collected and detected 4 WNV-positive *Cx. pipiens* mosquito pools from these traps in both areas, which represented the first positive mosquito pools in Sacramento County that year (*29*). Additionally, both human cases from Sacramento County with onset of illness before June 23 were located within cells identified by DYCAST as high risk, 12 and 2 days before onset of illness (Figure 3, June 23, 2005). Within Sacramento County, DYCAST predicted 142/152 (93.4%) of cases (11/163 cases were missing spatial or temporal data); 122/152 (80.3%) and 84/152 (55.3%) of cases were predicted 15 and 30 days, respectively, before onset of illness (Figure 4).

After 2005, the number of reported dead birds generally decreased (Table 1); the percentage of successfully geocoded dead bird reports ranged from 98.8% to 100% each year. The statewide DYCAST prediction rates for reported human cases during 2006–2009 were 26.3% (67/255), 33.0% (110/333), 16.3% (64/392), and 3.2% (3/93), respectively (Table 3). Responses to the 2006 and 2007 surveys were received from 47 agencies in 36 counties and 18 agencies in 19 counties, respectively. Results indicated that most of the agencies that responded each year used DYCAST to assist larviciding or adulticiding activities (Table 4). DYCAST prediction rates were significantly higher for agencies that answered “yes” to questions regarding larviciding (RR 10.06, 95% CI 2.45–41.32) and adulticiding (RR 10.91, 95% CI 2.65–44.88) in 2006 and larviciding (RR 10.16, 95% CI 1.41–73.00) in 2007 (Table 4). Conversely, the prediction rate was significantly lower for agencies answering “yes” to the question regarding adulticiding (RR 0.37, 95% CI 0.15–0.91) in 2007. However, excluding the most extreme outlier with respect to total number of cases, an agency in Kern County whose jurisdiction included 43 and 124 cases in 2006 and 2007, respectively, RRs were not significant in 2007 and were incalculable for 2006 (Table 4).

**Table 4:** DYCAST prediction rates of reported human West Nile virus cases, by survey answer, California, 2006–2007*

Year and activity	% (No.) agencies	Prediction rate				Prediction rate (excluding outlier)†		
		% Cases (no./total)	Rate ratio (95% CI)	p value		% Cases (no./total)	Rate ratio (95% CI)	p value
2006								
Larviciding			10.06 (2.45–41.32)	**0.001**			NA	NA
Yes	85.0 (34)	41.9 (52/124)				41.9 (52/124)		
No	15.0 (6)	4.2 (2/48)				0 (0/5)		
Adulticiding			10.91 (2.65–44.88)	**0.001**			NA	NA
Yes	74.4 (29)	39.7 (48/121)				39.7 (48/121)		
No	25.6 (10)	3.6 (2/55)				0 (0/12)		
2007								
Larviciding			10.16 (1.41–73.00)	**0.021**			5.63 (0.66–48.15)	0.115
Yes	72.2 (13)	56.4 (79/140)				31.3 (5/16)		
No	27.8 (5)	5.6 (1/18)				5.6 (1/18)		
Adulticiding			0.37 (0.15–0.91)	**0.031**			1.88 (0.22–16.05)	0.566
Yes	47.1 (8)	20.8 (5/24)				20.8 (5/24)		
No	52.9 (9)	56.4 (75/133)				11.1 (1/9)		

*DYCAST, Dynamic Continuous-Area Space-Time; CI, confidence interval; NA, not applicable. Agencies were asked whether they used DYCAST to assist larviciding, adulticiding; agencies that did not respond to survey, as well as answers of “Don’t know” (2006: larviciding: n = 2, adulticiding: n = 4) and missing data (2006: larviciding: n = 5, adulticiding: n = 4; 2007: adulticiding: n = 1), were omitted from analysis. Number of analyzed agencies with reported human cases: 2006: larviciding: “Yes”: n = 21, “No”: n = 4; adulticiding: “Yes”: n = 19, “No”: n = 7; 2007: larviciding: “Yes”: n = 6, “No”: n = 4; adulticiding: “Yes”: n = 5, “No”: n = 4. p values are 2 tailed; statistically significant associations (p<0.05) are in **boldface**. †Outlier, with respect to total number of cases, was a single agency with 43 and 124 cases in 2006 and 2007, respectively.

Discussion

Results from prospective implementation of the DYCAST system in California indicate that the risk model provided accurate and early identification of areas at high risk for WNV transmission to humans during a statewide epidemic in 2005, and was used by local agencies to assist public education campaigns, surveillance, and mosquito control programs. Our findings indicate that DYCAST yielded high levels of sensitivity and specificity for predicting human cases during the 2005 epidemic and that relative risk for a WNV case was >39× higher in high-risk cells than in low-risk cells (this value should be considered somewhat inflated, however, because not all low-risk cells contained populated areas). Given the low prevalence of cells containing cases (0.45%), the dynamic nature of DYCAST, and the (>1 cell) spatial scale of WNV transmission and mosquito control (*8*), positive predictive value is considered inferior to other metrics such as κ for evaluating model predictions. κ values >0.50 indicate that DYCAST correctly identified >50% of cells expected to be misidentified by chance alone, which is considered high because WNV causes symptoms in only ≈20% of infections (*28*). Values maintained a moderate strength of chance-adjusted agreement for >4 weeks before onset of illness, which indicates temporal robustness of model predictions.

**Figure 5:**
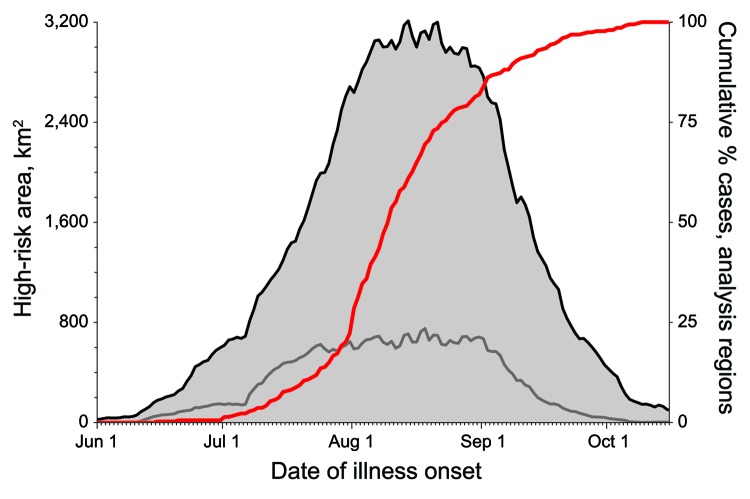
Analysis of West Nile virus cases, California, USA, 2005. Gray region represents area within all analysis regions (black line) and Sacramento County (gray line, for scale) designated by Dynamic Continuous-Area Space-Time as high risk by date of analysis. Red line represents cumulative percentage of reported human West Nile virus cases by date of onset of illness. Time between expansion of high-risk areas and subsequent increase in number of cases may provide an opportunity to respond before epidemic transmission occurs.

Cells containing predicted cases were identified as high risk before onset of illness by a mean of 37.2 days; given the 2–14 day range of the human WNV incubation period (*28*), this identification preceded transmission to humans and provided sufficient time to respond and potentially reduce the number of infections (Figure 4, Figure 5). Indeed, 252/354 (71.2%) of cases were predicted 15 days before onset of illness, before the maximum range of the incubation period. Additionally, because the DYCAST procedure only analyzes dead bird reports, it provided for more timely results than did active systems relying on the collection and testing of bird carcasses.

Results from Sacramento County in 2005 demonstrate the practical application of DYCAST for conserving and directing public health resources, such as targeting surveillance efforts that detected the county’s first positive mosquito pools that year. During subsequent months, Sacramento County was the location of the largest WNV epidemic in the United States, with 163 reported human cases (*30*) and an incidence rate of 14.5 infections per 100,000 population (*31*). DYCAST results played a key role in SYMVCD’s decisions for implementing and targeting emergency aerial mosquito control in the county (D. Brown, pers. comm.; 31), which ultimately reduced human illness and potential death from WNV infection (*32*).

Notably, prediction rates during 2006–2009 were substantially lower than in 2005, which has implications for the robustness of the model in nonepidemic years or regions. The fairly prevalent use of DYCAST results to assist mosquito control activities in 2006 and 2007 may have played a role in reducing the model’s prediction rates in circumstances in which WNV transmission was successfully interrupted before human infection occurred (*31**,**32*). However, while DYCAST could have helped to reduce the absolute number of cases, relatively higher prediction rates were generally observed for agencies that used DYCAST results compared with agencies that did not (Table 4). One explanation is that these areas may have had higher rates of WNV transmission initially, which in turn may have increased agencies’ likelihood of utilizing DYCAST for directing control activities or of simply conducting control activities in general. Furthermore, higher rates of WNV transmission may yield greater numbers of subsequent cases within high-risk cells or clusters, thereby increasing the prediction rate. This phenomenon could have also contributed to the higher prediction rates observed during the more severe epidemic in 2005, as could have the self-selecting nature of agencies that participated in the DYCAST program that year, which may have included areas with higher rates of WNV transmission compared with the rest of the state or to subsequent years.

Efficacy and sustainability of the DYCAST system may be compromised by declines in dead bird reporting, which could be caused by public fatigue or apathy, reductions in reporting infrastructure, or declines in bird deaths caused by herd immunity (*33*). Potential approaches for ameliorating these effects could include recalibration of DYCAST parameters (e.g., lowering the analysis threshold), strategic timing and targeting of press releases and media campaigns, and technologic solutions such as mobile phone application software and text messaging to disseminate information and facilitate the reporting of dead birds. Furthermore, it is uncertain how DYCAST results are affected by spatial and temporal heterogeneities of WNV transmission, including inter- and intraspecies variability in the competence (*21**,**34*), pathology (*6*), and distribution of vector and host populations (*35**,**36*). Other confounding factors may include demographic and socioeconomic composition of human populations (3*7*) as well as environmental (*38*) and meteorologic variation.

Regardless, DYCAST proved to be a timely and effective early warning system during a severe WNV epidemic. The use of such prospective measures enable the conservation and focus of valuable human and financial resources, which in some cases could be the difference in making an otherwise chaotic epidemic situation tractable. More responsive and efficient surveillance and control can prevent additional human disease, decrease reliance on more substantial control activities later in the season, and reduce indirect costs from medical expenses and productivity loss. The total cost of the 2005 WNV epidemic in Sacramento County alone has been estimated at ≈$3 million (*39*). Furthermore, dynamic monitoring of risk throughout the season may inform decisions for redirecting and triaging resources and may also provide a means for evaluating efficacy of mosquito control efforts. Ultimately, the DYCAST system illustrates the utility of establishing a biologically relevant, spatiotemporal framework for disease surveillance, and adaptation of the DYCAST method may be useful for detecting other infectious diseases and clustering phenomena.

This study also highlights the benefits of interdisciplinary and interagency collaboration; synergies between 2 academic institutions and a governmental public health agency shortened the time from research to implementation, and engagement with local mosquito control agencies enabled the practical application of results in real time. Furthermore, our findings demonstrate the potential of harnessing the public’s ability to provide timely and useful surveillance data through telephone and internet communications. The leveraging of similar sociotechnologic infrastructure, from mobile phones to internet search queries and social networks, may play a major role in the success, scalability, and cost-effectiveness of predicting and preventing emerging diseases in the future.
